# Professor Moslem Bahadori (1927–2022), a Matchless Role Model in Modern Academic Medicine in Iran

**DOI:** 10.34172/aim.2022.57

**Published:** 2022-05-01

**Authors:** Mohammad Hossein Azizi, Shahriar Dabiri, Shahin Akhondzadeh, Reza Malekzadeh

**Affiliations:** ^1^Academy of Medical Sciences of Iran, Tehran, Iran; ^2^Pathology and Stem Cells Research Center, Kerman University of Medical Sciences, Kerman Iran; ^3^Psychiatric Research Center, Roozbeh Hospital, Tehran University of Medical Sciences, Tehran, Iran; ^4^Digestive Diseases Research Center, Digestive Diseases Research Institute, Tehran University of Medical Sciences, Tehran, Iran

**Figure:**
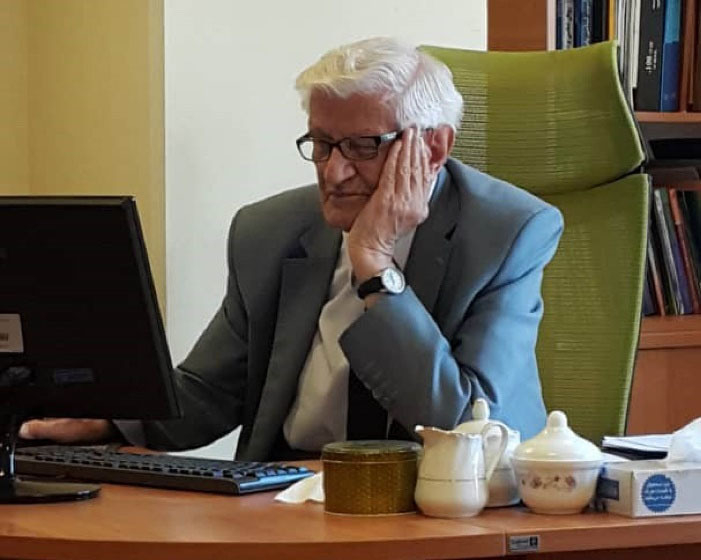
Professor Moslem Bahadori (1927–2022)

“*The mediocre teacher tells. The good teacher explains. The superior teacher demonstrates. The great teacher inspires.*” William Arthur Ward (1921–1994)

**With deep sadness, Dr. Moslem Bahadori (1927–2022), professor emeritus of pathology at the Tehran University of Medical Sciences, a superb mentor with brilliant ideas and a prolific researcher passed away on April 21, 2022 in Tehran. His demise was an immense loss not only for his family and friends, but also for the Iranian medical community and pathologists. Here, a brief account of his productive life and career is presented**.

 He was born in a large family on January 21, 1927 in *Zangisha Mahaleh*, a small village in the former Shahsavar city (now Tonekabon) in the Mazandaran Province, northern Iran. When he was a ten years old boy, he moved to his sister’s house in Tehran, where he flourished and completed his primary and secondary schools in 1948 (Figure 1).^1^

**Figure 1:**
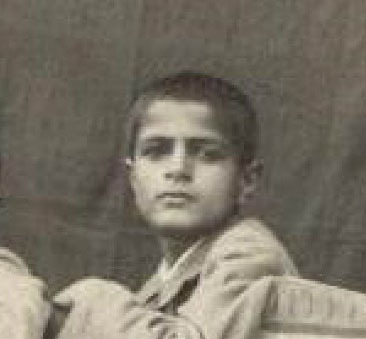
Professor M. Bahadori at the Age 10.

 He stated that, “In 1948, I participated in the entrance exam to the Faculty of Medicine, Dentistry and Pharmacy of the University of Tehran and became the twentieth volunteer among four thousand people”.^2^ Then, he enrolled at the Tehran University Medical School and obtained his MD diploma in 1954 (Figures 2-4).^1^

**Figure 2:**
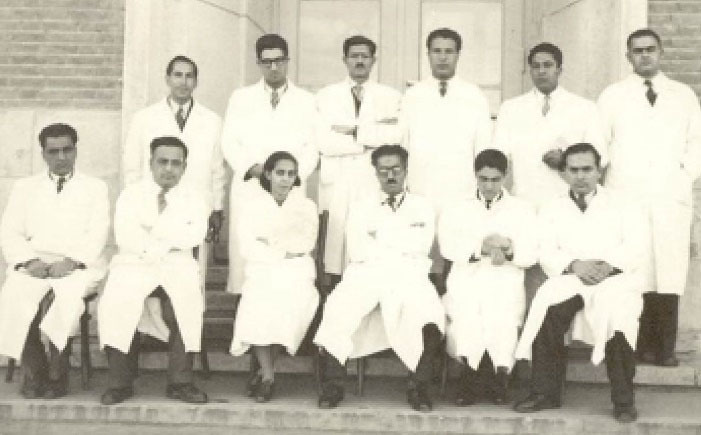
In the former Pahlavi Hospital during internship training, Professor Mohammad Gharib (1909–1975), the founder of modern pediatrics in Iran is sitting, third from right (1953).

**Figure 3:**
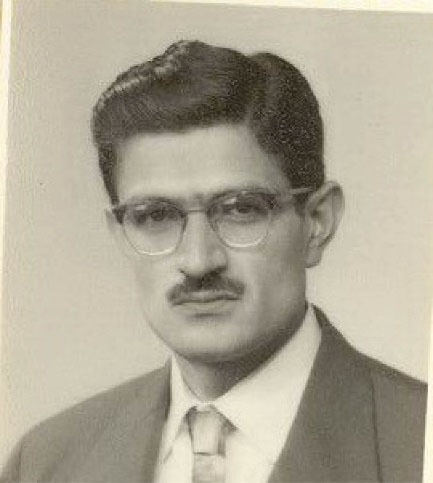
Professor M. Bahadori in his youth (1957).

**Figure 4:**
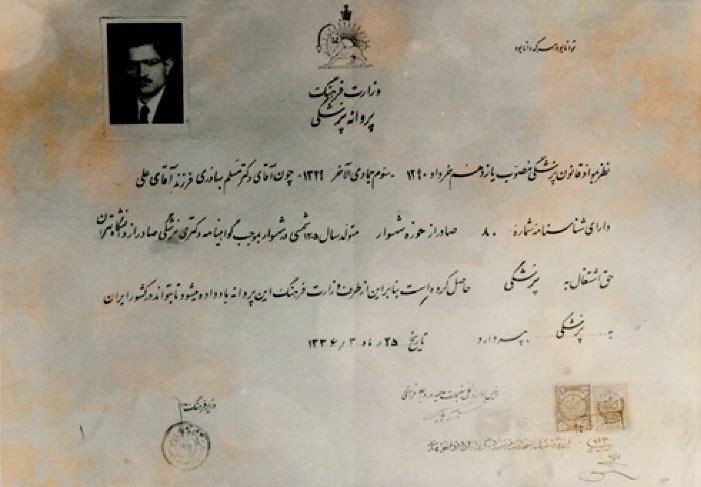
Dr. Bahadori MD Certificate (issued in 1957).

 In due course in 1955, the young Dr. Bahadori was accepted as a resident of pathology, when Professor Kamaleddin Armin (1914–1995) was the chairman of department. Dr. Bahadori became a pathologist in 1957 and joined that department as an assistant professor of pathology (Figure 5) and continued his training in cardiopulmonary pathology for 18 months in the Cardiff University, UK and became full professor in 1968 and then retired in 1997. He was the invited professor of cardiopulmonary pathology in the University of California in USA, twice in 1970–1971 and 1974–1975. Professor Bahadori was a fellow of the “American College of Chest Physicians” (Figure 6). ^1^

**Figure 5:**
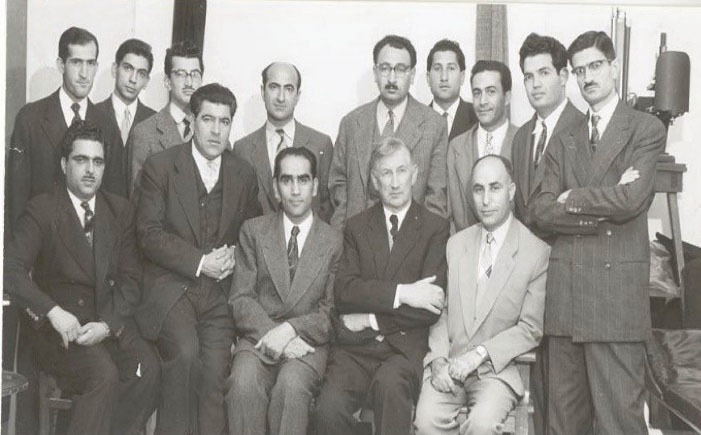
Professor M. Bahadori in the Central Pathology Laboratory. The first sitting row from right: Dr. Sajjadi, Dr. Krieberg, Dr. Armin, Dr. Habibi, Dr. Raees Bahrami (Tehran, 1969).

**Figure 6:**
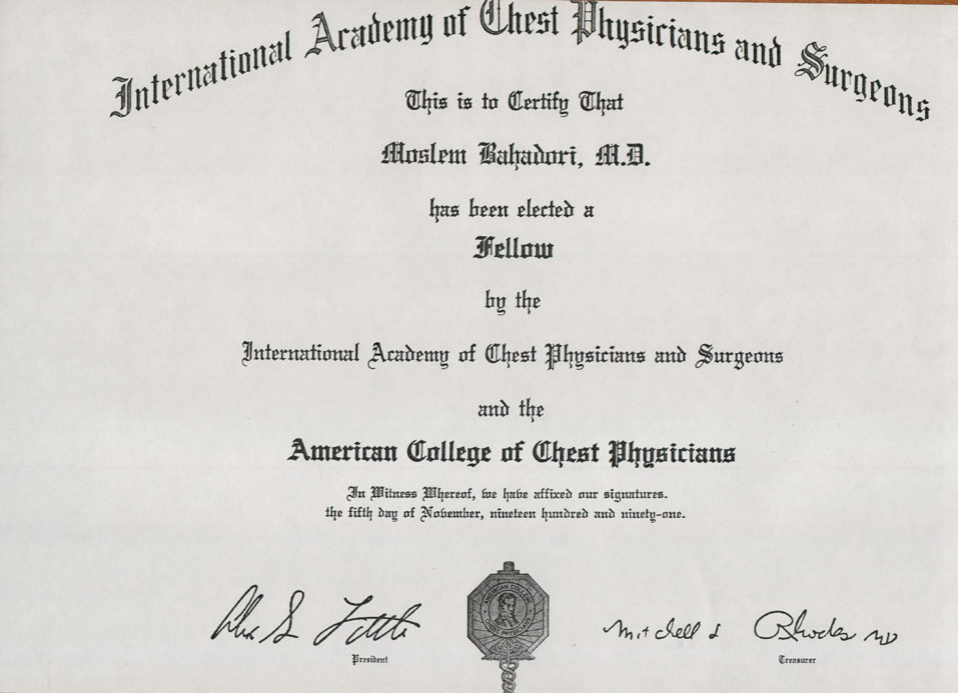
Professor Moslem Bahadori’ fellowship certificate of the “American College of Chest Physicians”, November 1991.

 Undoubtedly, he was one of the best medical professors in our country. He said in an interview, “I had no program in my life except reading, writing, teaching and learning, and even my administrative works have also been in these areas.”

 But what was the secret of his professional accomplishments that can be mentioned?

**First:** A pioneer in his field: Historically, the Medical School of University of Tehran was established in February 1934 and the pathology department was founded by Professor Mostafa Habibi Golpayegani (1904–1948). In 1939, When Dr. Charles Oberling (1895–1960), the French pathologist, became the Dean of medical school, the pathology residency training program was improved. The second generation of pathologists included Dr. Kamaleddin Armin (1914–1995) and Dr. Hossein Rahamatian, and the third generation were Dr. Abdul Mahmoud Shamsa and Dr. Mojtaba Sajjadi. Dr. Bahadori was among the fourth generation of pathologists in Iran.^3^

**Second:** His role in education: For decades; he had a crucial role in many medical students’ education and training of qualified pathologists in Iran. He taught pathology to medical and dentistry students and trained residents for 47 years, between 1957 and 2004.^1^

**Third:** Research, publication and participation in scientific meetings: He published his works in dozens of scientific articles in Persian and English (in total 133 articles until 2013) and authored 14 pathology textbooks in Persian from 1969 to 2009.^1^ In addition, for more than seven decades, he played a role in the promotion of Iranian medical journalism. Professor Bahadori’s presence in the field of medical journalism started when he was a second year medical student and cooperated with the first academic medical Persian journal named the “Monthly Letter of the Medical School” (the first issue was published in 1943) under the editorship of Dr. Nosratollah Kasemi (1909–1995), a professor of internal medicine. After that, in 1956, he collaborated with the first English-language medical periodical in Iran named “Acta Medica Iranica”, affiliated to Tehran Medical School under the editorship of Dr. Nosratollah Ameli (1913–2010). His efforts continued in other medical journals of the country until the last months of his life, including in the international journal of “Archives of Iranian Medicine, affiliated to the “Academy of Medical Sciences”, founded in October 1989 along with other colleagues. Over the years, he contributed greatly to the growth of the medical journals by participating in the editorial board of these periodicals, or as writer, a peer reviewer and scientific advisor (Figures 7-9).

**Figure 7:**
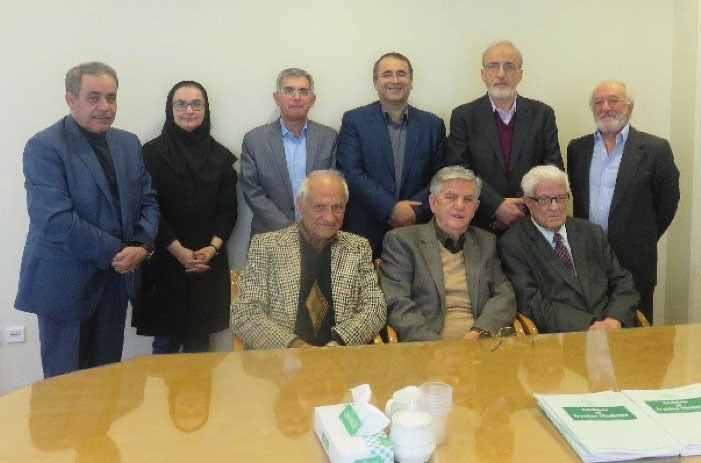
Professor M. Bahadori (sitting first from right) and his colleagues in the Archives of Iranian Medicine office.

**Figure 8:**
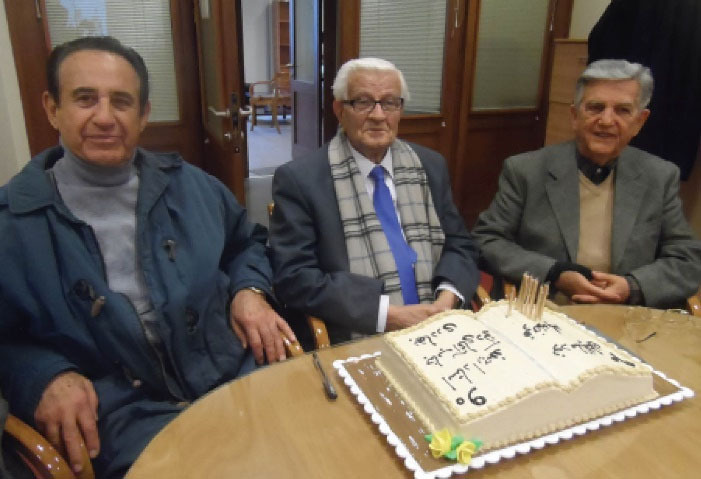
Professor M. Bahadori (sitting in the middle). His 90th birthday anniversary at the “Archives of Iranian Medicine Journal”. The late Professor Ismail Yazdi (1931–2020) and Professor Siavoush Sehhat are seen on the right and left sides, respectively.

**Figure 9:**
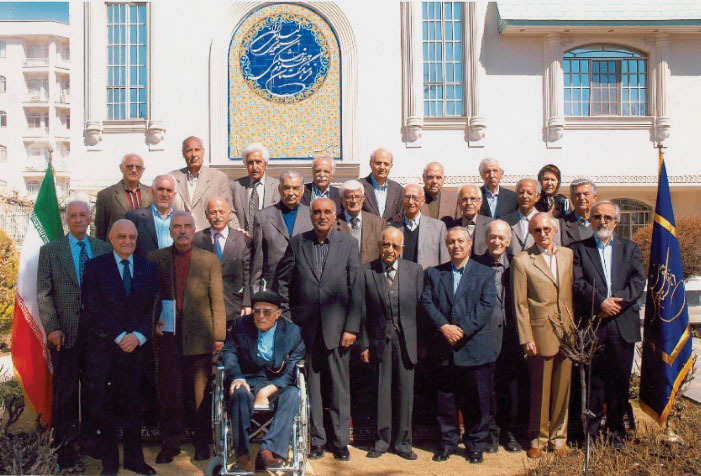
Professor M. Bahadori (standing, second row, fifth from right) and his colleagues, Academy of Medical Sciences.

 He actively attended many domestic and international scientific meetings, seminars and workshops (Figures 10 and 11).

**Figure 10:**
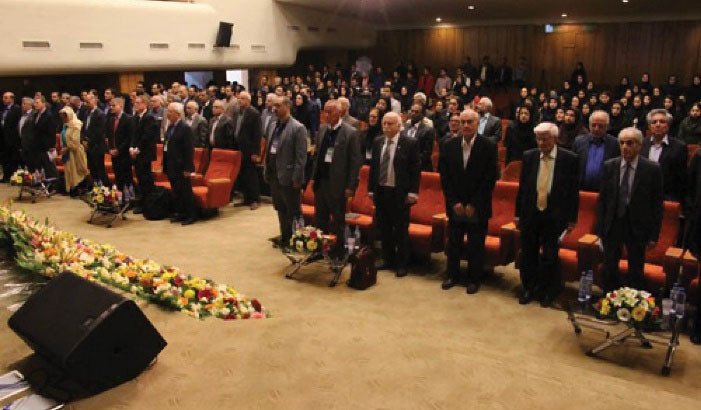
Professor M. Bahadori (standing, first row, second from right), at the first International Congress of Afzalipour Medicine with a focus on pathology, Kerman University of Medical Sciences, November 2015 (Photo by: Foad Ibrahimi, Public Relation of Kerman University).

**Figure 11:**
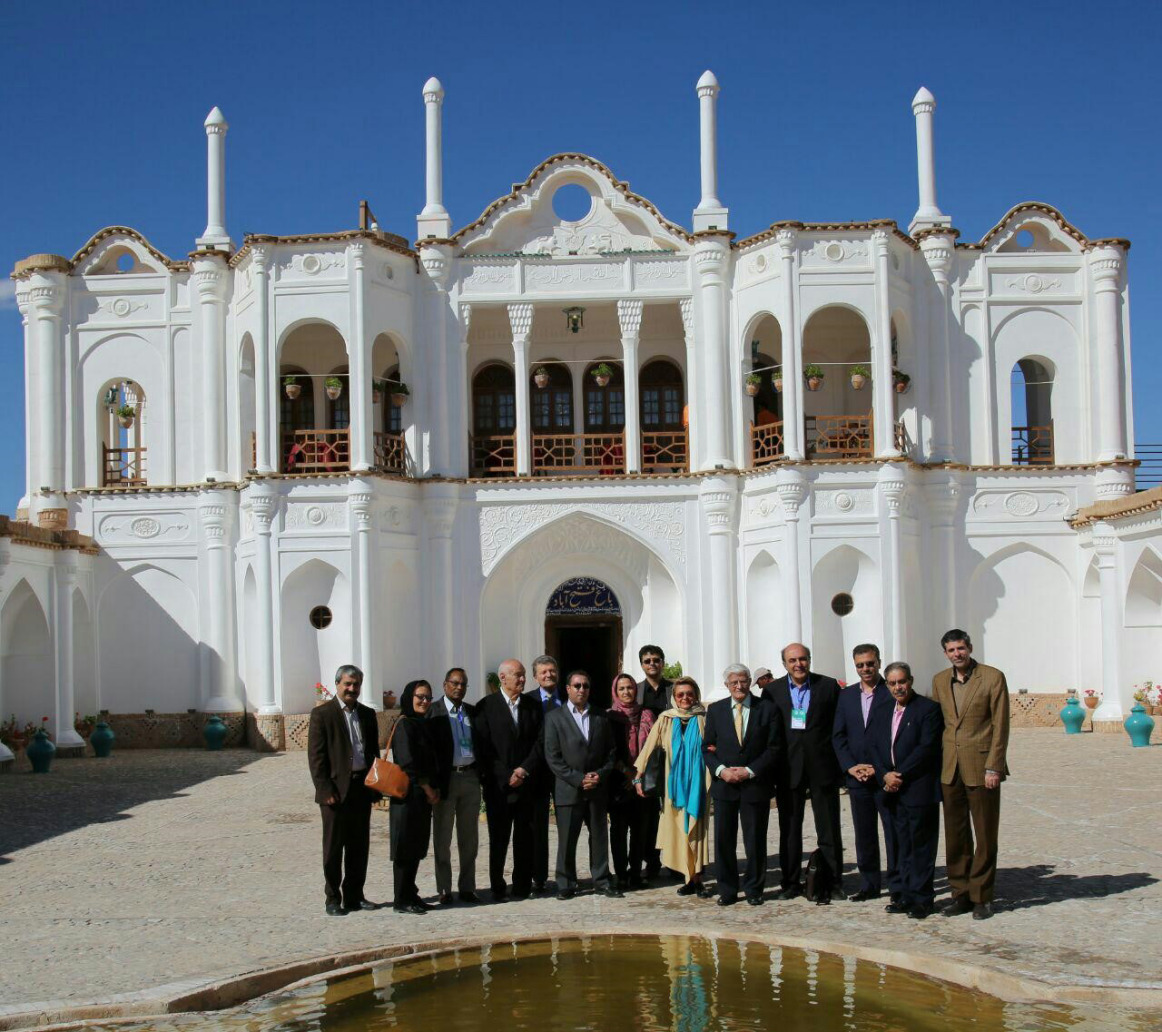
Professor M. Bahadori, fourth from right, Kerman, Iran, Fath Abad Garden, November 2015 (Photo by Public Relation of Kerman University).

**Fourth:** Some of his outstanding services: He was a founding member of the “Social Services Organization Council” (Dr. Rahnama Committee) in the early 1970s to assess the health and medical services in Iran.^1^ Finally, the results of this comprehensive assessment by the committee were published in February 1976, in a three-volume Persian book entitled “*Rahi Be Soui Tandrosty*” (A Way to Health), consisting of very informative opinions and inquiries from several clinicians and other medical and public health experts from various universities. In the introduction of the book, Professor Bahadori was mentioned as one of main contributors to that investigation (Figure 12).

**Figure 12:**
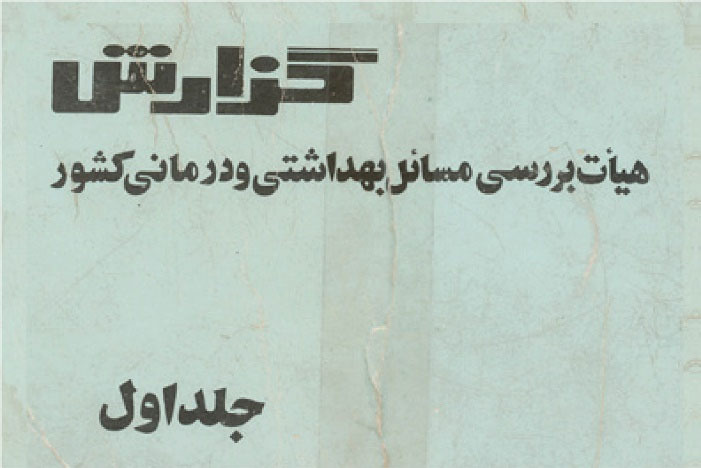
*Rahi Be Soui Tandrosty* (A way to Health) Vol.1, by Daruish Pirnia, Tehran, 1976.

 He was also one of the designers of the “National Health Network”, including Health Worker Training Program (*Tarbiat -e Behvarz*) as the frontier liners in the “Health Houses” in rural areas between 1972 and 1977.^1^

 Professor Bahadori started his scientific activities as a permeant member of the “Academy of Medical Sciences”, since its formal inauguration in the winter of 1990, and he became the “Head of the Basic Medical Sciences Group”. In addition, he was Head of the “Iranian Board of Pathology” for many years.

 He was an influential member of the “Iranian Association of Pathologists” and a founder and the first Head of the “Alumni Office of the Tehran University of Medical Sciences” established in 2006.^4^

 In addition, for the past five years, every year, he attended the “Congress of the Iranian Association of Pathologists”, named after him and Professor Parviz Dabiri (1921–2012), who was the founder of “Pathology Department at the University of Isfahan” and in that annual meeting, the top young researchers in pathology were awarded.^5^

**Fifth: **Professor Bahadori was a hardworking scientist who flourished through his perseverance and long-life efforts and has been influential in the development of modern medicine in Iran. The story of his life and career has been written in detail in his valuable diaries. The Persian autobiography of Professor Bahadori, named “*Afsaneh -ye Hasti*” (The Myth of Existence) was published in 2018, in 712 pages including 416 pages of text and 306 pages of images. It contains his recollections of the past decades of his life until 2017^1,6^ (Figure 13). The book is attractive for medical students, residents and physicians, as well as informative for non-medical book lovers who are interested in the history of contemporary Iran.

**Figure 13:**
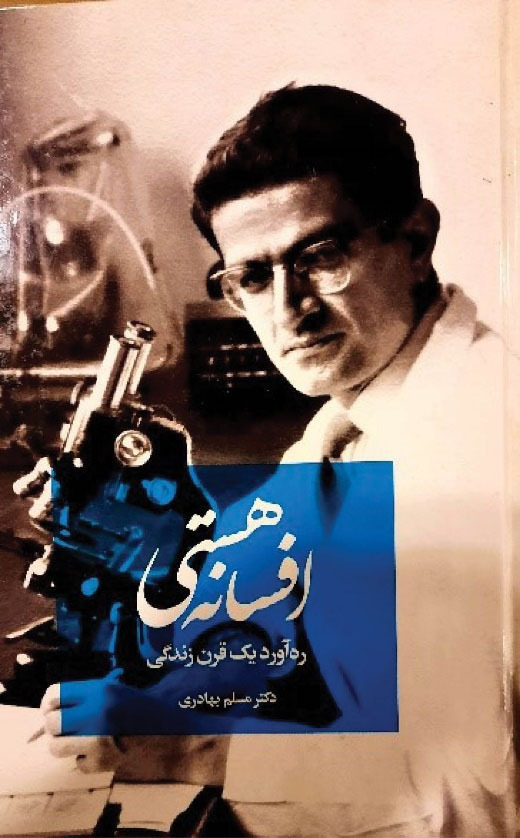
The Persian autobiography of Professor Bahadori, “*Afsaneh -ye Hasti*” (The Myth of Existence), 2018.

 In March 1961, he married Dr. Nahid Pishva (1933–1997), when she was a pathology resident. Later, in 1974, she became full professor of pathology at the department of pathology of Tehran University. At that time, she was the first lady physician who obtained her full professorship in Tehran University.^7^ His wife was a loyal companion to Dr. Bahadori and raised worthy children.

 Unfortunately, Professor Pishva passed away due to colon cancer on June 8, 1997 (Figure 14). From this marriage, they had three well-educated children who loved their father and took good care of him, especially in the last two years of his life, when he became ill and needed more care.

**Figure 14:**
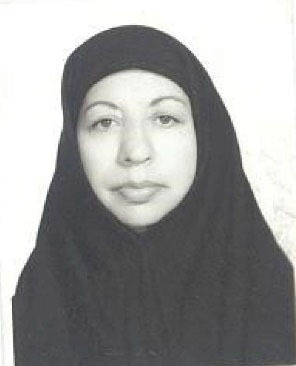
Dr. Nahid Pishva (1933–1997).^7^

 In summary, Professor Bahadori was a distinguished physician who was kind, generous, and always ready to help his family, patients and profession. He sincerely loved the culture, literature and history of his homeland and did not refrain from praising the true servants and honorable scientists and artists of Iran. He was tireless and hard-working. He loved books and reading and writing. His non-medical writings are as readable and instructive as his medical works. Professor Bahadori thought about the fate of all human beings and wished them well-being, health, justice and comfort. His knowledge in the field of medical science was always up to date and extensive and he had a bright memory. His presence was charming and his words were rich and all these placed Professor Bahadori among those who can be categorized as an excellent role model.

 Professor Bahadori passed away on Thursday, April 21, 2022. He was buried two days later in the “Famous People Sector” of Tehran cemetery. He is survived by his two daughters (Tina and Neda) and one son (Ali) and his grandchildren. His memory will not be forgotten, and he will always be remembered. Just in one word, Professor Bahadori was a symbol of hope in the future for our generation(Figure 15)^[1]^.

**Figure 15:**
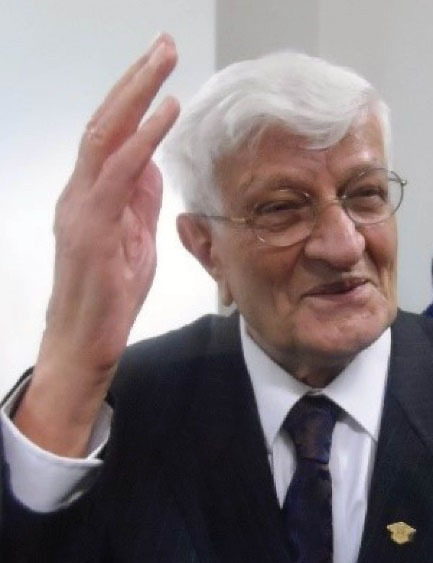
Professor Bahadori, 2016.

 Some of Professor Bhandari’s published papers in English in the last two decades of his life (2006–2022) are as follows^8^:

1. Azizi MH**, Bahadori M**. Professor Abdulkarim Vessal (1933-2022) and His Role in Promotion of Radiology and Medical Journalism in Iran. Arch Iran Med. 2022 Mar 1; 25(3):196-200.
2. **Bahadori M**, Azizi MH, Dabiri S, Bahadori M. Effects of Human Nucleolus upon Guest Viral-Life, Focusing in COVID-19 Infection: A Mini- Review. Iran J Pathol.2022 winter; 17(1):1-7.
3. Azizi MH, **Bahadori M**, Naseri M. Professor Fakhreddin Ghavami (1930-2021); a Role Model of Professional Commitment. Arch Iran Med. 2021 Nov 1; 24(11):858-861.
4. Azizi MH, **Bahadori M**. In Memory of Dr. Hooshang Zadanfarrokh (1937-2021), a Pioneering Iranian Otolaryngologist. Arch Iran Med. 2021 Oct 1; 24(10):786-787.
5. Azizi MH, **Bahadori M**. Dr. Touraj Nayernouri (1943-2021) and his fruitful academic life. Arch Iran Med.2021; 24(8):653-656.
6. Danaei G, Farzadfar F, Kelishadi R, Rashidian A, Rouhani OM, Ahmadnia S,Ahmadvand A, Arabi M, Ardalan A, Arhami M, Azizi MH, **Bahadori M**, et al. Iran in transition. Lancet. 2019 May 11; 393(10184):1984-2005
7. **Bahadori M**, Azizi MH, Dabiri S. Recent Advances on Nucleolar Functions in Health and Disease. Arch Iran Med. 2018 Dec 1; 21(12):600-607.
8. **Bahadori M**, Eslami M, Azizi MH. A Brief History of Oral and Maxillofacial Pathology in Iran. Arch Iran Med. 2018 Nov 1; 21(11):551-555.
9. Azizi MH, **Bahadori M**, Azizi F. An Overview of Epidemic Typhus in the World and Iran during the 19th and 20th Centuries. Arch Iran Med. 2016 Oct; 19(10):747-750.
10. Azizi MH, **Bahadori M**. The Five Decades of Academic Services of Professor Hassan Farsam (1932-2016) to Modern Pharmacy in Iran. Arch Iran Med. 2016 Jun; 19(6):453-4.
11. Azizi MH, **Bahadori M**, Dabiri S, Shamsi Meymandi S, Azizi F. A History of Leishmaniasis in Iran from 19th Century Onward. Arch Iran Med. 2016 Feb; 19(2):153-62.
12. Azizi MH, **Bahadori M**. Dr. Nasser Moeinzadeh (1930-2015); a Pioneering Otolaryngologist in Iran. Arch Iran Med. 2015 Oct; 18(10):737.
13. Azizi MH, **Bahadori M**, Dabiri S. Professor Kamaleddin Armin (1914-1995); a Superb Mentor with High Morals. Arch Iran Med. 2015 Oct; 18(10):729-33.
14. Azizi MH, **Bahadori M**, Dabiri S. Professor Parviz Haghighi and His Role in Promotion of Academic Pathology at the Shiraz School of Medicine in Iran (1969-1979). Arch Iran Med. 2015 Aug; 18(8):552-5.
15. Azizi MH, Nayernouri T, **Bahadori M**. The History of the Foundation of the Iranian National Blood Transfusion Service in 1974 and the Biography of its Founder; Professor Fereydoun Ala. Arch Iran Med. 2015 Jun; 18(6):393-400.
16. Azizi MH, **Bahadori M**, Dabiri S, Shamsi Meymandi S. Remembering Professor Amir Hossein Mehregan (1931-2000); the great Iranian dermatopathologist. Arch Iran Med. 2015 Feb; 18(2):139-42.
17. Azizi MH, **Bahadori M**. In remembrance of professor Shams Shariat Torbaghan (1926 - 2014). Arch Iran Med. 2014 Jul; 17(7):531-2.
18. Azizi MH, **Bahadori M**, Dabiri S, Shahpasandzadeh MH. In memory of the late Alireza Afzalipour, the founder of the Kerman University. Arch Iran Med. 2014 Jun; 17(6):457-60.
19. Azizi MH, **Bahadori M**, Azizi F. Breakthrough discovery of HbA1c by Professor Samuel Rahbar in 1968. Arch Iran Med. 2013 Dec; 16(12):743-5.
20. Azizi MH, **Bahadori M**, Azizi F. History of cancer in Iran. Arch Iran Med.2013 Oct; 16(10):613-22.
21. Azizi MH, **Bahadori M**. Brief historical perspectives of malaria in Iran. Arch Iran Med. 2013 Feb; 16(2):131-5.
22. Azizi MH, **Bahadori M**. The life and career of Professor Parviz Dabiri (1921-2012). Arch Iran Med. 2012 Oct; 15(10):657-8.
23. 23: Azizi MH, **Bahadori M**, Raees-Jalali GA. A historical profile of diphtheria in Iran during the 19th and 20th centuries. Arch Iran Med. 2012 Mar; 15(3):181-6.
24. Azizi MH, **Bahadori M**. A history of leprosy in Iran during the 19th and 20^th^ centuries. Arch Iran Med. 2011 Nov; 14(6):425-30.
25. Azizi MH, **Bahadori M**. A brief history of tuberculosis in Iran during the 19th and 20th centuries. Arch Iran Med. 2011 May; 14(3):215-9.
26. Azizi MH, **Bahadori M**, Raees-Jalali GA. In commemoration of Haj Mohammad Nemazee (1895-1972): the founder of Nemazee Hospital in Shiraz. Arch Iran Med.2009 May; 12(3):321-4.
27. **Bahadori M**, Azizi MH. The first medical journal of Tehran University. Arch Iran Med. 2007 Jul; 10(3):420-3.
28. Azizi MH, **Bahadori M.** In commemoration of Dr. Mostafa Habibi-Golpayegani (1904 - 1948): pioneer of modern pathology in Iran. Arch Iran Med. 2006 Oct; 9(4):438-41.

## Footnotes

**Endnotes**
[1] A few years ago, Professor Bahadori kindly gave the author (MHA), a number of his scanned old photographs, and diplomas along with a copy of his autobiography book, some of which appear in the current paper.
